# On the Emergence of Candida auris: Climate Change, Azoles, Swamps, and Birds

**DOI:** 10.1128/mBio.01397-19

**Published:** 2019-07-23

**Authors:** Arturo Casadevall, Dimitrios P. Kontoyiannis, Vincent Robert

**Affiliations:** a Department of Molecular Microbiology and Immunology, Johns Hopkins Bloomberg School of Public Health, Baltimore, Maryland, USA; b Division of Internal Medicine, The University of Texas MD Anderson Cancer Center, Houston, Texas, USA; c Westerdijk Fungal Biodiversity Institute, Utrecht, Netherlands; University of British Columbia

**Keywords:** *Candida*, climate change, fungus

## Abstract

The most enigmatic aspect of the rise of Candida auris as a human pathogen is that it emerged simultaneously on three continents, with each clade being genetically distinct. Although new pathogenic fungal species are described regularly, these are mostly species associated with single cases in individuals who are immunosuppressed. In this study, we used phylogenetic analysis to compare the temperature susceptibility of C. auris with those of its close relatives and to use these results to argue that it may be the first example of a new fungal disease emerging from climate change, with the caveat that many other factors may have contributed.

## Abstract (precis)

The most enigmatic aspect of the rise of Candida auris as a human pathogen is that it emerged simultaneously on three continents, with each clade being genetically distinct. Although new pathogenic fungal species are described regularly, these are mostly species associated with single cases in individuals who are immunosuppressed.

## OPINION/HYPOTHESIS

Candida auris is a new drug-resistant fungal species that was first isolated in 2009 from a human ear and thus named “*auris*” (1). Since then, C. auris has been associated with human disease in many countries, and the clinical isolates are remarkable for exhibiting nonsusceptibility to antifungal agents. One of the striking developments associated with the appearance of C. auris-related disease is that pathogenic isolates appear to have emerged independently on three continents simultaneously (2). Analysis of isolates recovered from the Indian subcontinent, Venezuela, and South Africa during 2012 to 2015 revealed that the isolates from each continent were clonal, but that those from different continents constituted genetically different clades (2). The mechanism(s) responsible for the simultaneous emergence of three different clades of C. auris in three geographically distant regions are unexplained.

The widespread use of antifungal drugs has been suggested as a contributory cause in the emergence of C. auris (3). Whereas selection by environmental azole use can certainly have contributed to drug resistance in this fungal species, it does not easily explain why this organism suddenly became a human pathogen on three continents. For example, the emergence of azole-resistant *Candida* spp. began long before the appearance of C. auris, and there does not appear to be a correlation between the emergence of azole-resistant *Aspergillus* spp. associated with azole agricultural use and the hot spots for C. auris emergence. The acquisition of drug resistance alone is very unlikely to confer upon a microbe the capacity for pathogenicity, since reduced susceptibility to drugs and virulence are very different properties, as evidenced by frequent fitness costs associated with mutations conferring resistance to antifungals to *Candida* (4, 5). Instead, as exemplified by the experience with Aspergillus fumigatus, aspergillosis was a well-known clinical entity before its putative acquisition of drug resistance from the agricultural use of azoles (6). Hence, one might have expected C. auris to have been known as a human and animal pathogen first and then to have acquired drug resistance, rather than emerging as drug resistant from agricultural drug use at the same time that it became a human pathogen. Another suggested explanation for the emergence of C. auris is that it recently acquired virulence traits that confer the capacity for virulence (7). Although this explanation cannot be ruled out, the capacity for virulence is a complex property that emerges from many attributes, and it is improbable that it would have occurred concurrently on three continents, unless driven by another factor that selected for it.

Human-pathogenic fungi constitute only a very small minority of an enormous number of fungal species in the environment, numbering in the few hundred (8). In contrast to ectothermic animals and plants, mammals are remarkably resistant to invasive fungal diseases. Mammalian resistance to invasive fungal diseases is proposed to result from a combination of high basal temperatures, which create a thermal restriction zone, and advanced host defense mechanisms in the form of adaptive and innate immunity (9). In this regard, the emergence of such thermodynamically unfavorable animals as mammals as the dominant large animals has been proposed to have resulted from a fungal filter at the Cretaceous-Tertiary boundary that prevented a second reptilian age (9). According to this theory, fungal pathogens are rare in mammals because this group of animals was selected by the fungi at the end of the Cretaceous period (9). Supporting this view are the facts that the majority of fungi grow well in ambient temperatures and that only a small percentage of species can replicate at 37°C (10). Consequently, invasive fungal infections are rare unless one of these two resistance pillars is disturbed. For example, the high prevalence of mycoses in individuals with advanced HIV infection was a result of a weakening of the immune system, while the white-nose syndrome in bats occurs during their hibernation process, when their temperature drops (11).

The thermal restriction zone that protects mammals is the difference between their high basal temperatures and the environmental temperatures. Human-induced climate change is anticipated to warm Earth by several degrees in the 21st century, which will reduce the magnitude of the gradient between ambient temperatures and mammalian basal temperatures (12). Consequently, there is concern that higher ambient temperatures will lead to the selection of fungal lineages to become more thermally tolerant, such that they can breach the mammalian thermal restriction zone. In this regard, experiments with an entomopathogenic fungus have shown that these can be rapidly adapted to growth at higher temperatures by thermal selection (13), establishing a precedent for an animal-pathogenic fungus adapting to mammalian basal temperatures. Most worrisome, analysis of thermal tolerances for fungal isolates deposited in a culture collection showed a trend of basidiomycetes for an increased capacity to replicate at higher temperatures, an observation consistent with an early adaptation to higher ambient temperatures beginning in the late 20th century (14). Supporting the notion of adaptation in response to temperature, fungal species in cities have become more thermotolerant than their rural counterparts (15). An analysis of the correlation of temperature tolerance with latitude (for strains isolated in recent decades and described in reference 14) showed that the Pearson correlations between the maximum temperature growth of all fungi, ascomycetes (e.g., *Candida* spp., *Aspergillus* spp., *Histoplasma* spp., etc.), and basidiomycetes (e.g., *Cryptococcus* spp., etc.) were −0.14619, −0.054638414, and −0.304251976, respectively. The lack of a significant trend for the ascomycetes is consistent with the fact that this group can grow at higher ambient temperatures and can thus grow across the latitudes. For the basidiomycetes, the borderline significant trend suggests that these are adapting to the warmer conditions in higher absolute latitudes, possibly because of climate warming, consistent with our earlier analysis (14). Given the capacity of fungal species to adapt to higher temperatures and the fact that many fungal species that are currently nonpathogenic species are likely to have the necessary virulence attributes by virtue of their survival in soils, we previously hypothesized that climate change would bring new fungal diseases (12).

**FIG 1:**
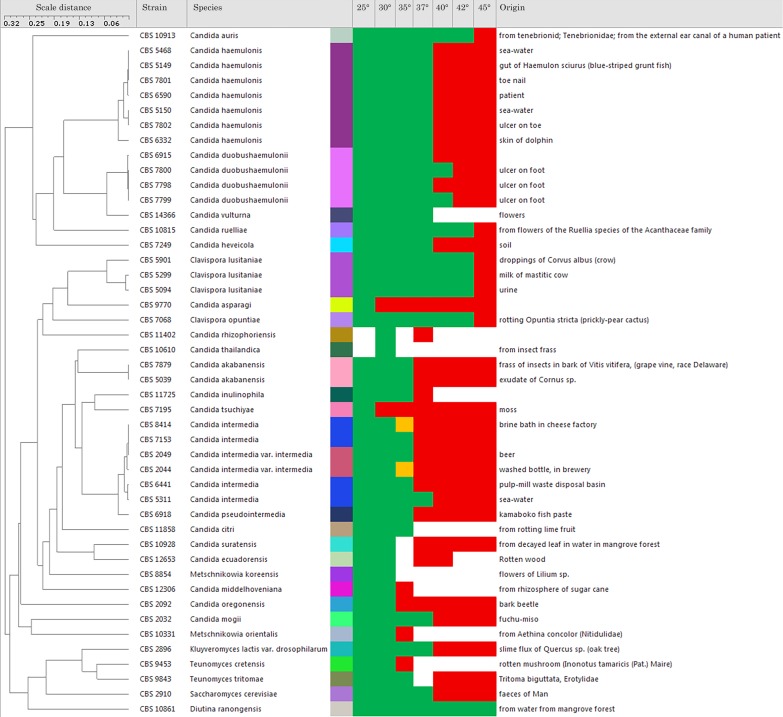
Comparison of thermal tolerance of C. auris and several close relatives. The tree shows that thermal tolerance is not monophyletic to closely related species. Green and red reflect temperature permissiveness and nonpermissiveness, respectively, for growth. *Candida* spp. with an upper limit of growth at 37°C are not be expected to grow at fever temperatures. Most closely related species manifest lower thermal tolerances than C. auris. The hierarchical tree (unweighted pair group method using average linkages [UPGMA]) is based on pairwise sequence alignments of both internal transcribed spacers and large subunit ribosomal DNA (data obtained and available from the CBS collection at www.westerdijkinstitute.nl).

C. auris is an ascomycetous yeast and a close relative of the Candida haemulonii species complex, which includes species occasionally pathogenic in humans and animals and demonstrates a high level of baseline antifungal drug resistance (16). This phylogenetic connection may explain its low susceptibility to antifungal agents and the possession of virulence attributes that confer it with pathogenic potential. To evaluate our hypothesis, we compared the thermal susceptibility of C. auris to those of some of its close phylogenetic relatives and found that the majority of these relatives were not tolerant for mammalian temperatures (Fig. 1). Although this tree reveals that C. auris is capable of growing at higher temperatures than most of its closely related species, it does not inform as to whether this is a new trait. It is noteworthy that the earliest description of C. auris came from a strain recovered from a human ear, which is much cooler than core body temperatures. Hence, this fungus may have gone through a short transient period during which it inhabited human surfaces before being associated with disease. Currently, C. auris preferentially colonizes the cooler skin rather than the hotter gut mycobiome, a preference that may be consistent with a recent acquisition of thermotolerance.

With this background, we propose the hypothesis that Candida auris is the first example of a new pathogenic fungus emerging from human-induced global warming. We posit that prior to its recognition as a human pathogen, C. auris was an environmental fungus. The fact that C. auris fails to grow anaerobically, along with the fact that it is typically detected on cooler skin sites but not in the gut, supports the notion that C. auris was an environmental fungus, until recently. Several factors, not necessarily mutually exclusive, may have been operative as to why C. auris emerged in the last decade. For example, as C. auris constitutively overexpresses HSP 90, this may account for its multidrug resistance, virulence, thermal tolerance, and osmotic-stress tolerance (17). Thus, C. auris might have previously existed as a plant saprophyte in specialized ecosystems, such as wetlands. As a first step, its emergence might have been linked to global warming (including climatic oscillations) effects on wetlands (18), and its enrichment in that ecological niche was the result of C. auris’s combined thermal tolerance and salinity tolerance. Interestingly, areas where C. auris was first recognized overlap, at least in part, the impacted wetland ecosystems (18). Of interest, C. albicans can be a part of a wetland ecosystem (19), and although many of the virulence determinants in the C. auris genome have not been characterized, it is theoretically possible that human-pathogenic *Candida* spp. have passed some virulence traits to previously nonpathogenic C. auris strains through plasmid DNA transfer (20, 21), in the backdrop of changing ecological niches. Alternatively, the effect of higher UV radiation in combination with global warming (22) might have contributed to mutagenic events that resulted in the suddenly increased fitness of a saprobe for survival in a host, via melanin- or non-melanin-dependent processes (23). C. auris’s jump from an environmental fungus to a fungus capable of transmission to, and pathogenic for, humans might have had an intermediate host, specifically an avian host, as fungi that can grow at 40 or 42°C can infect avian fauna. Of note, sea birds may serve as reservoirs for indirect transmission of drug-resistant *Candida* species, such as C. glabrata, to humans (24). The uncanny ability of *C auris* to adapt to specific niches, first in the environment and then in an avian host, might have led as a third step to its ultimate establishment as a human pathogen through genetic and epigenetic switches (25).

**FIG 2:**
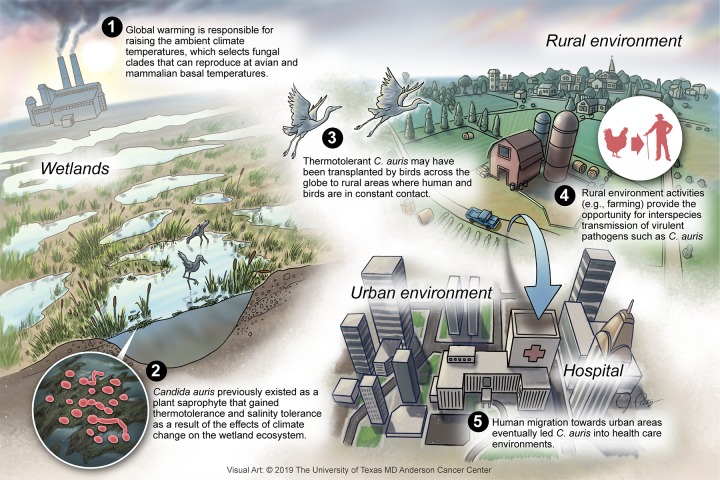
Proposed scheme for the emergence of C. auris.

The hypothesis that C. auris broke the mammalian thermal barrier through adaptation to climate change suggests several experimental lines of investigation to obtain evidence for and against it. If C. auris thermal tolerance is indeed a new property, a careful analysis of the temperature range that permits replication might reveal that it is still less tolerant than *Candida* spp. that have a long association with mammals. In this regard, isolates from earlier outbreaks may show differences in thermal tolerance from those of outbreaks that are more recent. Of course, analysis of thermal susceptibility requires a more detailed experimental approach to identify small differences than are normally measured by routine microbiologic characterization. A search for environmental reservoirs may produce closely related C. auris strains that have not yet adapted to higher temperatures, which may be expected if the process is stochastic and only some clades have made the transition. Increased sampling in these environments and hosts could test the involvement of wetlands and birds, respectively. Should evidence be found for C. auris or close relatives in these environments, then one might consider that the host jump from birds to humans follows mechanisms similar to those that operate for influenza virus. The possibility that C. auris was always a health care pathogen that has recently been discovered appears to have little support, given that no isolate was found prior to 1996 in fungal collections (3). Evaluating the contributory roles of high human population densities, migrations, increased city temperatures (15), poor hygiene, pollution, and regional and international travel (26) in the emergence of C. auris is difficult, given the available information, but these potential associations are fertile areas for research. The emergence of C. auris also poses interesting basic science questions on the thermal stability of its enzymatic processes and, in a broader sense, the mechanisms of virulence and adaptation for human-pathogenic fungi. For example, the evolutionarily conserved Hog1 stress-activated protein kinase (SAPK), the role for which in C. neoformans virulence is established (27), promotes stress resistance and virulence in C. auris (28), suggesting tantalizing connections between mechanisms for adaptation and virulence that are ripe for further study. A scheme for the possible factors operating on the emergence of C. auris is shown in Fig. 2.

Although global warming-related changes in the environment might have played a prominent role in C. auris’s emergence, this variable is unlikely to explain the whole story. For example, it is difficult to see how global warming alone explains the spontaneous emergence of four clades of C. auris strains in geographically disparate regions, each separated by thousands of years of evolutionary distance from each other, unless there is another common epidemiological variable that facilitated the interaction with humans for virulence to become apparent. Assuming that C. auris has other hosts in the biosphere, it is possible that climate change affects those hosts also and provides an additional variable for the emergence of this organism as a human-pathogenic fungus. We note that the recent clustering of cases in certain geographic regions, such as the northeastern United States, is a postemergence event that follows the proposed adaptation to climate change and probably reflects local infection control issues (29). Finally, we note that all four clades contain MTLa and MTLa loci (30), which raises the possibility that they may interact through mating if their current geographic isolation is ended by inadvertent human transport or migratory bird patterns.

Whether C. auris is the first example of new pathogenic fungi emerging from climate change or whether its origin into the realm of human-pathogenic fungi followed a different trajectory, its emanation stokes worries that humanity may face new diseases from fungal adaptation to hotter climates. Thus far, the majority of human cases of C. auris-related disease have occurred in debilitated individuals, such as those in intensive care units. Because their debilitated conditions impair their immunity, this group may serve as sentinels for the appearance of new fungal diseases. Perhaps the greatest lesson from the emergence of C. auris is the need for greater vigilance and continuous monitoring. In this regard, the environment is likely to contain large numbers of fungal species with pathogenic potential that are currently nonpathogenic for humans because they lack the ability to grow at mammalian temperatures. If anything, the direct and indirect effects of climate changes induced by an exponentially growing human population as drivers of fungal evolution should be an area of intense research in the decades to come. Widening of the geographic range of innately thermotolerant pathogenic fungi and the acquisition of virulence traits in thermotolerant nonpathogenic environmental fungi may shape the 21st century as an era of expanding fungal disease for both the fauna and flora of the planet.
